# Dental care use by immigrant Canadians in Ontario: a cross-sectional analysis of the 2014 Canadian Community Health Survey (CCHS)

**DOI:** 10.1186/s12903-019-0773-x

**Published:** 2019-05-08

**Authors:** Vrati M. Mehra, Christy Costanian, Siya Khanna, Hala Tamim

**Affiliations:** 1 School of Kinesiology & Health Science, York University, 4700 Keele Street, Toronto, ON M3J 1P3 Canada; 2 Department of Nutritional Sciences, University of Toronto, Toronto, Canada

**Keywords:** Oral health, Dental care use, Immigrants, Ontario

## Abstract

**Background:**

Ontario is home to the largest number of immigrants in Canada. However, very little is known about their dental care utilization patterns. The purpose of this study is to determine the prevalence of poor dental health care use among the immigrant population of Ontario and how various socio-demographic, socio-economic and health-related factors are associated with it.

**Methods:**

Analysis was performed on a total of 4208 Ontarian immigrants who participated in the dental care module of the 2014 cycle of the Canadian Community Health Survey. Poor dental care use was defined by the two variables: not visiting the dentist in the past year and/or visiting the dentist only for emergency purposes. Multivariable logistic regression was performed to assess the associations between the two outcomes and the socio-demographic, socio-economic and health-related factors.

**Results:**

Thirty three percent of immigrants reported not visiting the dentist in the past year and 25% reported visiting only for emergencies. The leading components associated with poor dental care utilization were being a new immigrant, of male gender, having low educational attainment, low household income and lacking dental insurance.

**Conclusions:**

This study is the first to highlight oral health care use patterns amongst immigrants in Ontario. Given that a large proportion of the immigrant population in Ontario have poor dental care use, education and outreach programs informing incoming immigrants of preventative dental care may improve overall dental health.

## Background

According to the 2016 Census, immigrants represented 21.9% of the entire Canadian population [1], a number that is projected to increase to 30.0% by 2036 [2]. With approximately 1,212,075 new immigrants deciding to permanently settle in Canada during years 2011–2016, the majority were from Asia and the Middle East (61.8%), followed by Africa (13.4%), Europe (11.6%), South and Central America (12.6%), and Asia Pacific rim countries (0.7%) [1]. As the rate at which the incoming immigrants contributing to Canada’s population increases, it becomes increasingly important to understand the needs and health status of this group. Doing so will help guide future health care policies and eventually build a healthier nation. As one of the largest provinces in Canada, Ontario is home to almost one-third of all immigrants in Canada [1]. However, despite such a large demographic, there is a lack of health-related data on immigrants residing in this province.

Regular dental care remains a cornerstone for both prevention and management of oral diseases, with adequate dental care utilization being reflected by periodic dental visits [3]. The Canadian Dental Association recommends that individuals partake in regular visits to the dentist every six months [3, 4]. Doing so can have many benefits for patients. Not only does regular oral health care ensure proper mental and social wellbeing [5–7], failure to prevent or control the progression of oral disease can increase the risk of adverse health outcomes, such as diabetes, cardiovascular disease, and cancer [8], all of which share common risk factors [8]. For example, bacterial infection in periodontal diseases increase oral inflammation, which in turn is associated with diabetes and cardiovascular diseases [9, 10].

Unfortunately, dental care use seems to be unequally distributed across populations [11–14]. After a period of 3 months following arrival, Canadian immigrants gain access to the publicly funded healthcare system, however, they do not receive access to oral healthcare as it is not included within the universal coverage [15]. The Canadian dental care system is almost exclusively private, due to this, significant disparities in its access exist [15–18]. Traditionally, low income and lack of dental insurance have been the primary barriers to access to dental care for all Canadians [16]. And while these factors also remain crucial barriers for immigrant Canadians’ access to dental care, they report worse oral health care utilization patterns than the general population [16, 19]. Moreover, there is a disproportionately higher amount of poverty reported amongst immigrants in comparison to their Canadian-born counterparts [20].

Newcomers may face unique challenges during the post-migration period that can significantly affect their oral health. Lower incomes, lack of awareness of oral health programs, as well as language barriers, are some of the obstacles that prevent immigrants from seeking proper dental care [15]. As a result of these challenges, immigrants in Canada seek other strategies to procure dental care. Transnational dental care is a popular strategy whereby immigrants seek dental care across national borders in the form of dental tourism and dental visits while travelling to their country of origin [21]. Calvasina et al., using data on over 3000 immigrants from the Longitudinal Survey of Immigrants to Canada (LSIC, 2001–2005), found that around 13% received dental care outside Canada over a period of 4 years [21]. This finding reflects on the inability of the current Canadian health care system to cater to the needs of diverse groups within its larger population. Given the role of culture and the burden of the immigration process on people’s perceptions of their oral health needs and social support seeking behaviors [22], more attention should be paid to the potential variations in dental care use by immigrants as compared to those born in Canada. Therefore, the aim of this study is to assess the characteristics associated with dental care use among Canadian immigrants residing in Ontario. Results from this study will help identify important factors associated with poor dental care use among the large demographic of immigrants residing in Ontario and will be useful for the planning of future policy interventions aimed at reducing the barriers in access to proper and timely care.

## Methods

### Study design, data collection and subjects

The present study was based on the 2014 Canadian Community Health Survey (CCHS), a cross-sectional survey conducted by Statistics Canada that gathers information on various health-related factors of Canadians. The survey data is comprised of a large sample of 65,000 respondents and helps inform reliable health-related information annually. The CCHS population includes individuals aged 12 and older residing across Canada. Excluded from the CCHS are those living on Aboriginal reserves, institutionalized populations, full-time Canadian Forces members, adolescents between the ages of 12–17 in foster care, and individuals residing in the Région du Nunavik and Région des Terres-Cries-de-la-Baie-James, in Quebec [23]. The survey uses multistage stratified cluster sampling strategy and all the responses to the survey are voluntary and collected via computer-assisted personal and telephone interviews [23]. For the purpose of this analysis, the study sample was limited to individuals aged 12 and above residing in Ontario. The survey provided complete information on an optional oral health module that captured information on dental care visit frequency, reason for dental visit and oral health habits, as well as who reported being immigrants.

Data were collected using the CCHS questionnaire designed for computer-assisted interviewing (CAI). The analysis was restricted to those who identified themselves as immigrants and resided in Ontario. Specifically, the results of the dental visit and oral health modules were used. These modules were part of the ‘optional content’ and were chosen by the province of Ontario to be a part of the survey for that year [23].

### Outcome

Dental care utilization comprised two variables: time since last dental visit and reason for visiting the dentist. Time since last dental visit was measured based on the following: “When was the last time that you went to a dentist?” For this question respondents were provided with the following options: “less than a year”, “1 year to less than 2 years ago”, “2 years to less than 3 years ago”, “3 years to less than 4 years ago”, “4 years to less than 5 years ago”, “5 or more years ago” and “Never”. The responses were recoded as last dental visit < 1 year (regular) or ≥ 1 year (irregular) [24]. The second outcome, reason for visiting the dentist was determined by the question: “Do you usually visit the dentist for emergency care or only emergency care?” For this question, the participants were provided with the following options: “more than once a year for checkups”, “about once a year for checkups”, “less than once a year for checkups”, and “only for emergency care”. These were categorized as either for checkups only or for emergency only. To encompass both outcomes when interpreting the results, poor dental care use in this study is defined as infrequent visits to the dentist and/or only emergency visits to the dentist [24].

### Covariates

The following self-reported variables were adjusted for as confounders of dental care use amongst immigrants: Socio-demographic factors: Years since immigration (categorized as < 10 years,10–20 years and > 20 years), age (categorized as < 18, 18–34, 35–54 and ≥ 55), sex (categorized as female and male), marital status (categorized as partner and no partner).

Socioeconomic status: Educational level (categorized as University education, High school diploma /College and Less than high school diploma), household income (categorized as $100,000–$150,000 and over, $30,000- < $100,000 and < $30,000), current work status (categorized as yes and no), dental insurance (categorized as insured and not insured).

Health-related factors: Smoking status (categorized as no and yes), alcohol use (categorized as Never, Less / At least once per month, At least once per week), health of teeth and mouth (categorized as Excellent/Very good, Good, Fair/poor), Perceived general health (categorized as Excellent/Very good, Good, Fair/poor), stress in life (categorized as Not at all stressful and Somewhat/extremely stressful).

### Statistical analysis

Descriptive statistics of the main outcomes and the covariates were conducted. A chi-square test was performed to assess the proportion of those who had each of the two outcomes. Binary Logistic Regression was conducted and the unadjusted ORs along with their 95% Confidence Intervals (CIs) were reported. Multivariable logistic regression analysis was then conducted and the adjusted ORs and 95% CIs obtained. This was done to adjust for all the covariates and obtain associations between each covariate and poor dental care use. Additionally, due to the survey’s complex sampling design, bootstrapping was performed to calculate the 95% CI estimates. Population weights, normalized weights and bootstrap weights were created by Statistics Canada and provided along with the CCHS file and were used in order to account for non-response bias. The analysis was performed using Stata Data Analysis and Statistical Software (Stata, version 13.0). The Alpha level was set at 0.05 for a two-tailed test.

## Results

The sample consisted of 4208 subjects representing 3,736,500 people, where 33.2% reported not having visited the dentist in the past year and 25.1% reported visiting the dentist only for emergencies. Table 1 presents characteristics of the subjects and the association between the covariates and poor dental care use. Approximately 51% of the subjects were females and 49% were males. More than 40% of the subjects lacked dental insurance coverage. Almost 60% of the subjects had a college or university education.

**Table 1:** Characteristics of the study sample and of the two outcomes (past year dental visits and emergency visits to the dentist for immigrants in Ontario

Variables	Total N^a^ (%)	Dental Visits ≥ 1 year	Emergency visits
		Unadjusted OR (95%CI)	Unadjusted OR (95%CI)
Weighted Total		1,240,500 (33.2%)	937,900 (25.1%)
Socio-Demographic Factors			
Years since immigration			
< 10 years	816,900 (22.1)	**1.90 (1.40–2.57)**	**1.52 (1.10–2.10)**
10–20 years	896,100 (24.2)	1.12 (0.84–1.50)	1.12 (0.80–1.60)
> 20 years	1,990,800 (53.8)	1	1
Age (years)			
< 18	151,500 (4.1)	1	1
18–34	792,200 (21.2)	**4.74 (2.44–9.20)**	1.66 (0.74–3.69)
35–54	1,319,400 (35.3)	**2.60 (1.34–5.01)**	0.85 (0.39–1.84)
≥ 55	1,473,400 (39.4)	**3.21 (1.69–6.10)**	**1.78 (1.78–3.89)**
Sex			
Female	1,901,100 (50.9)	1	1
Male	1,835,300 (49.1)	**1.40 (1.10–1.79)**	**1.37 (1.04–1.82)**
Marital Status			
Partner (ref)	2,490,900 (33.1)	1	1
No Partner	1,233,700 (66.9)	1.19 (0.94–1.51)	**1.48 (1.13–1.95)**
Socio-economic status			
Education Level			
University education	2,186,100 (59.5)	1	1
High school diploma/College	863,600 (23.5)	1.39 (0.98–1.95)	**1.69 (1.21–2.36)**
Less than high school diploma	622,200 (17)	**1.96 (1.44–2.68)**	**3.12 (2.26–4.31)**
Household Income			
$100,000–$150,000 and over	986,300 (26.4)	1	1
$30,000- < $100,000	2,091,900 (56.0)	**2.66 (1.90–3.80)**	**3.49 (2.39–5.09)**
< $30,000	658,300 (17.1)	**6.78 (4.60–10.10)**	**11.04 (7.16–17.03)**
Current Work status			
Yes	2,102,600 (56.3)	1	1
No	1,633,800 (43.7)	**1.35 (1.10–1.70)**	**1.95 (1.46–2.60)**
Dental Insurance			
Insured	2,023,100 (56.9)	1	1
No insurance	1,531,000 (43.1)	**4.98 (3.85–6.45)**	**5.89 (4.36–7.96)**
Health Related Factors			
Smoking Status			
No	3,300,400 (88.3)	1	1
Yes	435,400 (11.7)	**1.69 (1.13–2.54)**	1.52 (0.99–2.34)
Alcohol Use			
Never	1,335,700 (35.8)	1	1
Less/At least once per month	1,334,300 (28.4)	**0.72 (0.55–0.94)**	**0.64 (0.46–0.87)**
At least once per week	1,057,500 (35.8)	**0.58 (0.43–0.79)**	**0.43 (0.31–0.60)**
Health of teeth and mouth			
Excellent/Very good	1,735,800 (47.8)	1	1
Good	1,236,800 (34.1)	1.32 (1.00–1.80)	**1.47 (1.09–1.99)**
Fair/poor	659,400 (18.2)	**1.86 (1.40–2.60)**	**3.14 (2.22–4.44)**
Perceived General health			
Excellent/Very good	2,067,000 (55.4)	1	1
Good	1,140,500 (30.6)	1.32 (1.00–1.80)	**1.58 (1.21–2.07)**
Fair / poor	521,500 (14.0)	**1.70 (1.20–2.40)**	**2.38 (1.64–3.46)**
Stress in life			
Not at all stressful	507,600 (13.6)	1	1
Somewhat/ extremely stressful	3,214,700 (86.4)	0.99 (0.73–1.36)	0.91 (0.66–1.27)

^a^ Frequencies are row percentages estimated using normalized weights

Unadjusted odds ratios (ORs) and 95% confidence intervals (CIs) reported. Results from the Canadian Community Health Survey (CCHS), 2014

The bolded refect statistically significant findings

The results of the multivariable logistic regression have been presented in Table 2. Those who immigrated less than 10 years ago and between 10 and 20 years ago were more likely to visit the dentist for emergency procedures, as compared to those who immigrated more than 20 years ago, (OR: 2.02, CI-1.17-3.50; OR:1.92, CI-1.18-3.13), respectively. Individuals who were older than 18 years of age were at increased odds of not having visited the dentist in the past year and usually visiting for emergencies purposes. Likewise, males were 1.84 times (95% CI: 1.35–2.51) more likely to not have visited the dentist in the past year and 2.02 (95% CI: 1.42–2.89) times more likely to go for emergency dental visits compared to females. Socioeconomic status was associated with dental care use, where those who lack high school education and earn less than $100,000 per year were significantly more likely to have poor dental care use. Furthermore, immigrants without insurance were significantly at greater odds of having poor dental care use compared to those with insurance. Lastly, those who rated their oral health as fair or poor had greater odds of visiting the dentist for emergencies (OR = 1.92, 95% CI: 1.19–3.11). On the other hand, immigrants who consumed alcohol at least once per week had a significantly reduced odds of poor dental care use (OR = 0.67, 95% CI: 0.45–0.98; OR = 0.53, 95% CI: 0.35–0.81, respectively.

**Table 2:** Multivariable logistic regression analysis for past year dental visits and emergency visits to the dentist

Variables	Dental Visits ≥ 1 year	Emergency Visits
	Adjusted OR (95% CI)	Adjusted OR (95%CI)
Socio-Demographic Factors		
Years since immigration		
< 10 years	1.51 (0.94–2.41)	**2.02 (1.17–3.50)**
10–20 years	1.23 (0.83–1.84)	**1.92 (1.18–3.13)**
> 20 years	1	1
Age (years)		
< 18	1	1
18–34	**17.67 (6.75–46.26)**	**4.09 (1.23–13.54)**
35–54	**12.47 (4.39–35.43)**	3.59 (1.00–12.92)
≥ 55	**10.84 (3.72–31.59)**	**6.00 (1.69–21.26)**
Sex		
Female	1	1
Male	**1.84 (1.35–2.51)**	**2.02 (1.42–2.89)**
Marital Status		
Partner	1	1
No Partner	0.97 (0.70–1.35)	1.15 (0.80–1.64)
Socio Economic Status		
Education Level		
University education	1	1
High school diploma/College	1.01 (0.68–1.51)	1.25 (0.84–1.88)
Less than high school diploma	**2.06 (1.32–3.24)**	**2.31 (1.44–3.70)**
Household Income		
$100,000–$150,000 and over	1	1
$30,000- < $100,000	**1.96 (1.30–2.96)**	**2.47 (1.59–3.83)**
< $30,000	**4.38 (2.67–7.16)**	**5.74 (3.30–10.01)**
Current Work status		
Yes	1	1
No	0.78 (0.55–1.10)	0.84 (0.55–1.29)
Dental Insurance		
Insured	1	1
No insurance	**4.36 (3.19–5.95)**	**4.44 (3.16–6.22)**
Health Related Factors		
Smoking Status		
No	1	1
Yes	1.62 (0.97–2.71)	1.41 (0.80–2.48)
Alcohol Use		
Never	1	1
Less than/At least once per month	**0.69 (0.49–0.96)**	0.69 (0.47–1.03)
At least once per week	**0.67 (0.45–0.98)**	**0.53 (0.35–0.81)**
Health of teeth and mouth		
Excellent/Very good	1	1
Good	1.02 (0.71–1.47)	1.21 (0.86–1.71)
Fair/poor	1.07 (0.72–1.59)	**1.92 (1.19–3.11)**
Perceived General health		
Excellent/Very good	1	1
Good	1.13 (0.80–1.61)	1.30 (0.91–1.86)
Fair/poor	1.32 (0.83–2.09)	1.30 (0.79–2.14)
Stress in life		
Not at all stressful	1	1
Somewhat/ extremely stressful	1.09 (0.77–1.55)	1.09 (0.74–1.60)

The odds ratios (ORs) and 95% confidence intervals (CIs) reported. Results of the Community Health Survey (CCHS), 2014

The bolded refect statistically significant findings

## Discussion

This present study analyzed the association of various socio-demographic, socioeconomic and health-related factors, as well as poor dental care use within the immigrant population of Ontario. Almost 33.2% immigrants reported not visiting the dentist in the past year compared to 27.8% Ontarians [25]. Over a quarter of immigrants reported visiting the dentist for emergencies only in comparison to 19.3% of Ontarians [25]. The results further demonstrated that socio-demographic factors such as newly immigrating to Canada, being older than 18 years of age and being male were associated with poor dental health care utilization. Moreover, socio-economic factors such as low educational attainment, low household income and no dental insurance were also associated with poor dental health care use. Understanding the factors related to dental care utilization is key to decreasing the burden of poor oral health among immigrants in Ontario.

This study showed that recent immigrants i.e. those who immigrated less than 10 years ago and those who immigrated 10–20 years ago were significantly more likely to utilize emergency dental services than those who immigrated more than 20 years ago. This finding is in line with a study conducted by Newbold and Patel that showed that recent immigrants are more likely to visit the dentist only for emergency dental procedures such as fillings and extractions, compared to the native-born Canadians, who were more likely to visit the dentist for preventative care [15]. A possible explanation for this finding is that incorrect beliefs about dental health status might be preventing some groups from seeking treatment in a timely manner, therefore compelling them to seek emergency care upon the worsening of symptoms. In fact, a study conducted in Nova Scotia found that 45% of the recent adult immigrants rated their oral health as good, very good or excellent, despite having untreated decay and periodontitis [17]. This further illustrates the discrepancy that can exist between one’s perceived oral health and their actual oral health. The current study also revealed that immigrants older than 18 years of age, especially those aged 18 to 34 years were significantly more likely to have poor dental care use. This result is similar to the trends observed by the National Hospital Ambulatory Medical Care Survey (NHAMCS) from 1997/98 to 2007/08, that showed a significant increase in the number of emergency dental visits for the total population, even when the population growth was taken into account [26]. In addition, a study using nationally representative data from England and Wales, United States, Japan, and Sweden demonstrated that oral health levels usually decline as age increases. In the study they noted that dental caries’ levels and subjects’ DMFT index (the number of decayed, missing, and filled permanent teeth) increased with increasing age [27]. Sex was another important covariate in the present study, with males having greater odds of reporting poor dental care use than females. These differences are in accordance with previous studies which have shown that females are more likely than males to visit a dentist regularly [15] and practice positive health behaviors such as brushing their teeth twice a day [28].

Socioeconomic status indicators were significantly associated with poor dental care use in the present study. Immigrants with low household income were at increased odds of having poor dental care use. This result is consistent with the fact that dental care access and utilization for the overall adult population in Canada is highly dependent on personal income and the availability of insurance coverage [25, 29–33]. This finding is also in concordance with a study conducted by Calvasina et al. [34] on Brazilian immigrants in Toronto, Canada, where it was found that those with an average family income of < 30,000 CAD per year were almost four times more likely to not have visited a dentist within the previous year. In the current study, the association between education level and dental care use was also significant, whereby those with high education level were less likely to report poor dental care use. Education can be a means through which people can learn about the underlying mechanisms of disease development and the need for preventive services, which in turn could act as a catalyst in making people heed more attention to their oral health [34–36].

In the current study, a greater likelihood of poor dental care use was also observed among immigrants with no dental insurance. Given that in Canada the primary source of financing dental care is through employment-based dental insurance [18, 32], this result highlights the importance of increasing access to dental care for uninsured and underserved individuals. Since these individuals are required to pay out of their own pockets for their dental services, cost-prohibitive barriers might be preventing them from seeking regular dental care and ignoring symptoms until they require emergency attention. The promotion of health equality has been a well-established in Canada’s approach to health promotion, however as many as a third of all paid workers in Ontario lack employment based dental insurance [37]. While province wide and somemunicipal led subsidized programs exist for children and adults aged 65 years and older respectively [38, 39] those who lie outside these age brackets, regardless of their income status, are excluded from these benefits. Thinly sliced categories, although beneficial to some, hide the reality of a major portion of the population that is still left bereft of affordable dental care.

Health-related factors such as alcohol use and general health perceptions were also linked with oral health care utilization. The present study showed that alcohol had a protective effect on dental care utilization in the immigrant population, which has been previously reported using the same dataset [25]. In another study conducted in the United States, a positive association between light to moderate alcohol use and maintenance of one’s natural teeth was also observed [40]. Research has shown that alcohol use can be affected by acculturation levels. Acculturation is the process of learning and change that occurs when individuals from one culture are exposed to different cultural settings [41]. Agic et al. in their study on alcohol consumption among immigrants in Ontario, found that the patterns of alcohol use in the immigrants’ country of origin played an important role in predicting their alcohol consumption upon their arrival to Canada [41]. While acculturation levels can modulate health behaviors to a certain degree, including seeking timely dental care, due to the lack of information on their country of origin, it is difficult to assess how one’s culture mediated the participants’ alcohol use and dental care patterns in the present study. Finally, people reporting their oral health as fair/poor were significantly more likely to seek emergency care. This is in concordance with previous studies that show that Canadians suffering from worse health outcomes are less likely to seek preventative dental care and tend to rely more on emergency dental care services [29].

To our knowledge, this study is the first to examine socio-demographic, socio-economic and health-related factors with respect to dental care use in the immigrant population in Ontario. Although many factors that were associated with poor dental use among immigrants are similar to those among the general population in Ontario [25], this study shows that immigrants have much greater odds of having poor dental care use, as compared to their Canadian born counterparts. This observation is in line with previous research in Canada, which shows that immigrants although face similar barriers in access to dental care to the general population, but are affected more severely by these barriers than the general population [16, 29]. However, some limitations should be mentioned. Due to the cross-sectional nature of this study, causality cannot be inferred. All variables, including the two main outcomes, are self-reported, and hence subject to information bias. A measurement based on a clinical examination would have been more objective and accurate. Future research should incorporate variables such as cultural beliefs about oral health, family network and support. Furthermore, system-level variables should also be incorporated because individuals’ oral health status and dental care use may be based on their province [42]. Given that this study is a secondary analysis, no information regarding other dental health variables such as the number of decayed/missing/ filled teeth, oral pain, flossing habits, and depth of periodontal pockets was available. Lastly, given the heterogeneous and dynamic nature of the immigrant population, it is possible that the results of this study may not be generalizable to all immigrants. Hence, the authors recommend that further studies exploring specific dental care patterns and needs in different cultural groups within the immigrant population be conducted. Nonetheless, the study had several strengths. This study utilized a relatively large sample size allowing for ample statistical power, with population weights accounting for nonresponse bias. Confounding bias was also minimized due to the variety of potential covariates that were controlled for in the analysis.

## Conclusion

This study is the first to highlight oral health care use patterns among immigrants in Ontario. The estimate of not visiting the dentist within the past year was over 30%, while 25% reported visiting for emergency purposes only. Males, new immigrants, those with low household income, low educational level, no dental insurance, and poor health of teeth and mouth were at an increased odds of poor dental care use. It is vital that the provincial government and its stakeholders modify policies to support this population with their oral health care needs. Given the importance of immigration-specific factors, strategies and interventions to provide individuals with access to dental care at an early stage of immigration should be sought. This can be achieved via educational and outreach programs informing incoming immigrants of the importance of preventative dental care and where to find dental services in their region. Furthermore, appropriate use of language assistance and navigation services can be helpful in improving accessibility and use of these services. Lastly, publicly funded financial support programs for immigrants without dental insurance should be implemented. Doing so will not only improve dental care seeking behaviors, but also help create a healthier nation by preventing chronic conditions that often follow dental diseases.

## Abbreviations

CCHS: Canadian Community Health survey
CI: Confidence interval
OR: Odds ratio
